# Characterizing Environmental Surveillance Sites in Nigeria and Their Sensitivity to Detect Poliovirus and Other Enteroviruses

**DOI:** 10.1093/infdis/jiaa175

**Published:** 2020-04-09

**Authors:** Abdullahi Walla Hamisu, Isobel M Blake, Gerald Sume, Fiona Braka, Abdullateef Jimoh, Habu Dahiru, Mohammed Bonos, Raymond Dankoli, Ahmed Mamuda Bello, Kabir M Yusuf, Namadi M Lawal, Fatimah Ahmed, Zainab Aliyu, Doris John, Theresa E Nwachukwu, Michael F Ayeni, Nicksy Gumede-Moeletsi, Philippe Veltsos, Sidhartha Giri, Ira Praharaj, Angeline Metilda, Ananda Bandyopadhyay, Ousmane M Diop, Nicholas C Grassly

**Affiliations:** 1 World Health Organization Nigeria, Abuja, Federal Capital Territory (FCT), Nigeria; 2 Department of Infectious Disease Epidemiology, Imperial College London, London, United Kingdom; 3 National Primary Health Care Development Agency, Garki, Abuja, FCT, Nigeria; 4 Public Health Development, Garki, Abuja, FCT, Nigeria; 5 WUPA Wastewater Treatment Plant, Abuja, FCT, Nigeria; 6 World Health Organization Regional Office for Africa, Cité du Djoué, Brazzaville, Republic of Congo; 7 Novel-t Sàrl, SATIGNY, Geneva, Switzerland; 8 Division of Gastrointestinal Sciences, Christian Medical College, Vellore, India; 9 Bill & Melinda Gates Foundation, Seattle, Washington, USA; 10 World Health Organization Headquarters, Geneva, Switzerland

**Keywords:** environmental, eradication, poliovirus, sewage, surveillance

## Abstract

**Background:**

Environmental surveillance (ES) for poliovirus is increasingly important for polio eradication, often detecting circulating virus before paralytic cases are reported. The sensitivity of ES depends on appropriate selection of sampling sites, which is difficult in low-income countries with informal sewage networks.

**Methods:**

We measured ES site and sample characteristics in Nigeria during June 2018–May 2019, including sewage physicochemical properties, using a water-quality probe, flow volume, catchment population, and local facilities such as hospitals, schools, and transit hubs. We used mixed-effects logistic regression and machine learning (random forests) to investigate their association with enterovirus isolation (poliovirus and nonpolio enteroviruses) as an indicator of surveillance sensitivity.

**Results:**

Four quarterly visits were made to 78 ES sites in 21 states of Nigeria, and ES site characteristic data were matched to 1345 samples with an average enterovirus prevalence among sites of 68% (range, 9%–100%). A larger estimated catchment population, high total dissolved solids, and higher pH were associated with enterovirus detection. A random forests model predicted “good” sites (enterovirus prevalence >70%) from measured site characteristics with out-of-sample sensitivity and specificity of 75%.

**Conclusions:**

Simple measurement of sewage properties and catchment population estimation could improve ES site selection and increase surveillance sensitivity.

## Teaser

Easily measured environmental surveillance site characteristics, including sewage properties recorded with a water-quality probe, predict site sensitivity to detect poliovirus and other enteroviruses. Data collection during site selection could identify better sites and improve the sensitivity of global poliovirus surveillance.

Surveillance for poliovirus relies on the detection and reporting of cases of acute flaccid paralysis (AFP), with isolation and sequencing of poliovirus from stool required to confirm diagnosis of poliomyelitis. However, only approximately 1 in 1000 poliovirus infections results in AFP, and the majority of (asymptomatic) infections are thus not detected, allowing “silent” transmission of infection.

Poliovirus is shed in stool for 6 weeks on average during asymptomatic infection and may be detected in sewage or wastewater contaminated with fecal material [1, 2]. In populations with convergent sewage networks, testing of sewage for poliovirus can therefore be a more sensitive method of detecting virus circulation than AFP surveillance [3–5]. This approach, referred to as environmental surveillance (ES), relies on collection of sewage using a single bucket “grab” sample or occasionally more sophisticated methods (eg, bag-mediated filtration, composite sampling), virus concentration (eg, 2-phase separation, filtration), and detection (typically, growth in cell culture).

Recognizing the benefits of poliovirus ES as a supplement to AFP surveillance, the Global Polio Eradication Initiative (GPEI) developed a global ES expansion plan for 2013–2018 [6]. At the end of 2018, the GPEI supported over 45 countries conducting poliovirus ES compared with just a handful before the implementation of this plan [7]. Expanded ES has played a crucial role in the eradication effort, from detection of circulating vaccine-derived poliovirus (VDPV) outbreaks in Africa and Asia to identification of wild-type poliovirus spread across Pakistan [8, 9].

The sensitivity of ES to detect poliovirus circulation in a given population depends on the nature of the sewage network, the appropriateness of the sampling site, and the quality of sample handling and laboratory processing [5, 10]. High sensitivity is critical to allow timely detection of outbreaks and to ensure absence of detection is indeed evidence for absence of circulation. The global expansion of poliovirus ES has been rapid with heterogeneous implementation, resulting in between 3 and 120 sites per country undergoing regular (typically monthly) sample collection. Isolation of oral vaccine (Sabin) poliovirus after vaccination campaigns has shown considerable variability among sites, perhaps reflecting variation in campaign coverage but also variation in their sensitivity to detect poliovirus [11]. Isolation of nonpolio enteroviruses (NPEVs) is also routinely reported and is expected for almost all ES samples given the high prevalence of these viruses among children in low-income countries [12]. Nonpolio enteroviruses are affected by dilution and inactivation effects in sewage in a manner similar to poliovirus. Absence of any enterovirus (poliovirus or NPEV) detection is therefore indicative of poor ES sensitivity and can be used to identify poor performing ES sites that should be targeted for investigation or closure [13]. However, it typically takes at least 1–2 years before a new site is identified as inappropriate based on enterovirus detection, leading to wasted resources and gaps in surveillance.

Current GPEI guidelines recommend establishment of ES sites where there is a convergent sewage network and a catchment population of 100 000 to 300 000 people [14]. However, most areas at high risk of poliovirus transmission have informal drainage and sewerage arrangements for which catchment areas are documented poorly or not at all. Even if the catchment area can be defined, reliable data on population numbers are not available at this geographic scale in most ES countries. This makes estimation of the catchment population difficult and identification of suitable ES sampling sites challenging.

To improve ES site selection and sensitivity, we conducted a study in Nigeria during 2018–2019 to measure ES site characteristics and determine their association with the isolation of human enteroviruses including poliovirus. Our findings inform the next generation of GPEI guidelines for poliovirus ES and are relevant to ES for other pathogens such as typhoid.

## METHODS

### Environmental Surveillance Site Investigation

Five field teams, each consisting of 1 World Health Organization (WHO) and at least 1 national government staff member, made quarterly visits to ES sites across Nigeria, with each team allocated sites in 3 to 5 states after a training workshop in Abuja. Power calculations indicated that to identify an association between a single ES site characteristic and “good” site performance (defined as a prevalence of enterovirus isolation >70%) with 80% power and assuming a large effect size (Cohen’s d = 0.8), we would need to visit 50 sites, assuming half were good and a 5% significance level. If there was an imbalance in the proportion of sites with good performance (eg, 2:1), this number increased to 59, and for smaller effect sizes further increases in the number of sites were required. Therefore, we planned to visit all 78 ES sites with regular sample collection in Nigeria at the time of study planning (May 2018). At each site, latitude, longitude, and altitude were recorded using a GPS device with ±10-meter accuracy and a photograph of the sampling location was taken. Characteristics of the site on the day of the field team visit were reported using an electronic questionnaire hosted on a mobile phone using Open Data Kit (ODK). Variables recorded were speed of sewage flow, direction of flow, depth and width, color, smell, and open or covered drainage channel. Answers were selected from predefined categories.

After completing the questionnaire, the field team recorded water quality parameters from the sewage sampling site using an Aquaprobe AP-2000 with an optional optical turbidity meter included (Aquaread Ltd., Broadstairs, Kent, UK). Parameters recorded included temperature, pH, oxidative reductive potential (ORP), dissolved oxygen, total dissolved solids (TDS), salinity, and turbidity. A protocol for safe and accurate deployment of the water quality probe was developed in advance of the study after pilot testing at the Christian Medical College, Vellore, India. This includes rapid calibration of the probe before visiting the ES site, probe sanitization after use, and instructions on appropriate personal protective equipment. Each field team was allocated a water quality probe, and all probes underwent a full calibration before each quarter of data collection. At least 2 readings were taken at each site visit, and the average of these readings was used in the statistical analysis.

Environmental surveillance officers in each state completed an electronic survey at the beginning of the first round of data collection using a mobile ODK application. Survey questions included the date the ES site began operation, usual frequency of sample collection, whether sewage flow varied during the day or seasonally, estimated catchment population and method of estimation, and presence of local public services or infrastructure from a predefined list (schools, transit or commercial hubs, hospitals, or health facilities, factories) and their distance from the site (walking time). We also obtained catchment population estimates from the GPEI ES Site Catalogue, which is based on watershed estimates from digital elevation models (DEM) and synthetic and field-collected streams and waterways combined with GRID3 GIS-based population estimates at a 90-meter resolution [15]. In addition, we estimated the population living within 2 km of each ES site based on their GPS location and publicly available WorldPop 2015 population data for Nigeria at 100-meter resolution [16].

### Laboratory Data

We included laboratory data for ES samples collected between June 1, 2018 and May 31, 2019. Environmental surveillance sample characteristics on arrival in the laboratory are routinely recorded, including the time of sample collection, temperature of the sample carrier, time taken to arrive in the laboratory, sample condition and volume, concentrate volume, and time taken from arrival in the laboratory to inoculation in cell culture. The laboratory algorithm for cell-culture detection of poliovirus and NPEVs in ES samples is described in detail elsewhere [14, 17].

### Statistical Analysis

Quarterly data from the field teams were collated together with the ES officer survey data and the laboratory database for individual ES samples. To analyze the association between quarterly data on ES site characteristics and results from individual samples, each sample was matched to site data collected during the quarter corresponding to the date of sample collection (eg, Q1 data collected in August 2018 was used for samples collected during June–August 2018, etc).

We analyzed quarterly variation in ES site characteristics within and between sites using analysis of variance and assessed linear correlation between variables using Pearson’s correlation coefficient. We used mixed-effects logistic regression to determine the association of site characteristics with enterovirus detection (poliovirus or NPEV). We included a random effect by site to account for repeat observation and a random effect over time (cyclic monthly random walk) to allow for seasonal trends in circulation of enteroviruses, dividing the country into 3 zones by latitude (Sahel in the north, Savanna in the middle, and Guinea in the south [18]). We used this model to investigate univariable associations with enterovirus detection and subsequently selected a multivariable model using forward stepwise regression based on the widely applicable information criterion (WAIC). In the multivariable model, we compared models that included the 3 different catchment population estimates and chose the final model based on the WAIC. Continuous variables were transformed into categorical variables with 3 levels corresponding to the lower quartile, interquartile range (IQR), and upper quartile. The models were implemented in the R-INLA package [19] using the R statistical programming language [20].

We subsequently aggregated enterovirus and ES site characteristic data for the entire study period and used machine learning (random forests) to determine whether site characteristics were able to predict good sites (enterovirus prevalence >70%) versus “bad” sites (enterovirus prevalence ≤70%) [21]. We aggregated water quality parameters across the 4 quarterly measurements by calculating the mean temperature and pH, minimum ORP, and dissolved oxygen and maximum TDS and turbidity. In this way, we sought to reflect measurements most likely to correspond to high levels of fecal contamination measured during at least 1 visit. We also examined the predictive ability of just a single (quarter 1) measurement of site characteristics and water quality data. We used 10-fold cross-validation repeated 20 times to determine out-of-sample predictive accuracy using the randomForest and crossval packages in R [22, 23].

## RESULTS

### Environmental Surveillance Site Characteristics

**Figure 1.**
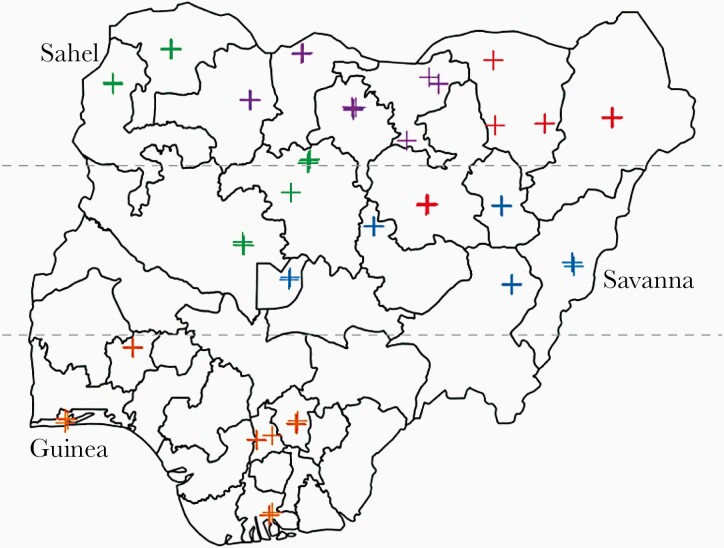
Location of poliovirus environmental surveillance (ES) sites included in the study based on GPS readings from the quarterly visits of each field team. Locations are indicated by a cross and shaded according to study team (n = 5). The dashed lines are plotted at latitudes defining the 3 climate zones used in the statistical analysis, defined as Guinea (coast-8°N), Savanna (8–11°N), and Sahel (11–16°N) following Omotosho and Abiodun 2007 [18]. Note that at this scale, the crosses for neighboring ES sites may overlap because of their proximity.

**Figure 2.**
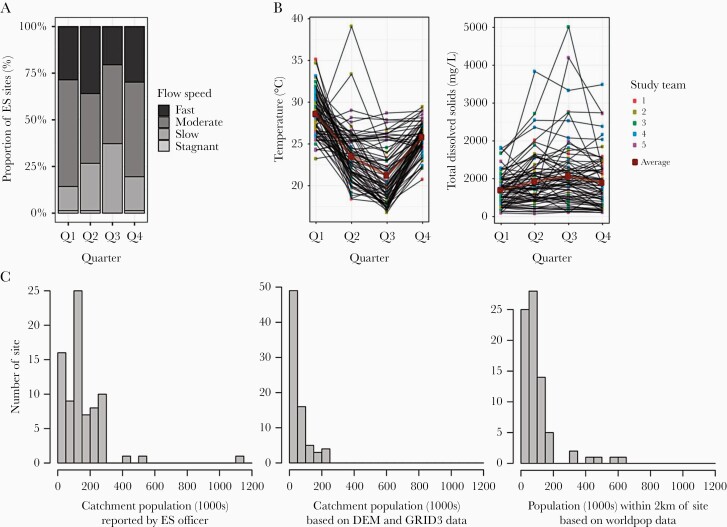
Environmental surveillance (ES) site characteristics. Quarterly variation in (A) sewage flow rate recorded in the electronic ES field team survey and (B) sewage temperature and total dissolved solids measured using the water quality probe. (C) Distribution of ES site catchment population estimates based on the ES officer survey, digital elevation models (DEM)/mapping from Novel-t or WorldPop estimates of the local population within a 2-km radius. In B, lines connect measurements at the same site over time, points are shaded by study team, and the average across all measurements each quarter is shown by the thicker line. Quarter refers to study quarter (ie, Q1 is for data collected in August 2018, etc).

Seventy-eight ES sites were visited by the field teams in all 21 states with poliovirus ES at the time of commencing the study (Figure 1). Four visits were made at every site during the following periods: August 8–23, 2018; November 7–20, 2018; January 23–February 8, 2019; and April 16–June 5, 2019. Measurements were taken in the morning when ES samples are also usually collected, on average at 8:35 am (IQR, 6:55 am to 9:05 am). Environmental surveillance site characteristics collected by the field team including water quality parameters showed some seasonal variation, depending on the measurement (Figure 2). However, with the exception of temperature, water quality parameters all showed significantly more variation between ES sites than within a site over time (F-statistic 2.26 to 648, *P* values all <.001) (Table 1). Sewage flow rate reported by the field team showed significant seasonal variation and was slower during the third quarter, January–February 2019, corresponding to the dry season (χ ^2^ test, *P* = .0258). Sewage depth and width were usually reported as deep (54.9%) and wide (74.7%) and did not show significant variation by quarter (χ ^2^ test; *P* = .436 and .714, respectively). A smell of sewage was reported during 88.3% of ES site visits.

**Table 1.** Summary of ES Water Quality Probe Measurements by the Field Team Including Results of an ANOVA for Variation Between Sites Versus Within Sites Over Time

Variable	Mean (IQR)	F Statistic	*P* Value
temperature (°C)	24.8 (21.8–27.1)	0.733	.945
pH	7.8 (7.6–8.1)	3.835	<.001
Oxidative reductive potential (mV)	−58.5 (−197.8 to 77.2)	3.609	<.001
Dissolved oxygen (%saturation)	55.9 (37.7–74.8)	2.925	<.001
Total dissolved solids (mg/L)	898.2 (434.2–1170)	7.134	<.001
Turbidity (NTU)	57 (11.9–61.1)	2.259	<.001

Abbreviations: ANOVA, analysis of variance; ES, environmental surveillance; IQR, interquartile range; mV, millivolts; NTU, nephelometric turbidity units.

The results from the ES officer survey indicated site initiation dates between 2011 and 2018 (mode 2016). The majority of sites were reported to have daily (52 of 78) or seasonal (66 of 78) variation in sewage flow, with increased flow reported in the mornings and during the rainy season. Twenty-two percent (17 of 78) of ES sites reported at least 1 hospital or health facility within a 10-minute walk (mean number of hospital or health facilities 1.2 among those reporting at least 1). Eighty-three percent (65 of 78) reported at least 1 primary or secondary school (mean 3.0), 67% (52 of 78) reported at least 1 transit or commercial hub (mean 2.2), and 21% (62 of 78) reported at least 1 factory (mean 2.4) within a 10-minute walk (means are for those sites reporting at least 1).

Catchment population size estimates reported by ES officers were based on local vaccination campaign “microplans” (39 of 78), census data (30 of 78), DEM (5 of 78), or an approximation (4 of 78). These catchment population size estimates did not correlate significantly with estimates based on DEM/GRID3 (Pearson’s correlation coefficient r = 0.22, *P* = .0542) or the population within 2 km based on WorldPop (r = −0.20, *P* = .0779). Environmental surveillance officer estimates of catchment population size were larger on average than those based on DEM/GRID3 (median size 117 000 vs 26 500) (Figure 2). Digital elevation models/GRID3 catchment population estimates showed a modest correlation with the population within 2 km based on WorldPop (r = 0.28, *P = .*0145). Catchment population estimates showed limited correlation with water quality parameters (Supplementary Figure 1).

### Enterovirus Isolation

**Figure 3.**
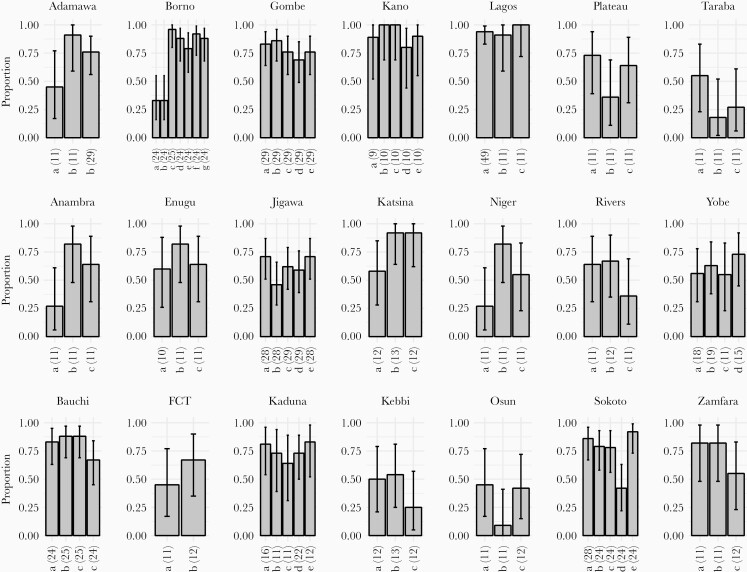
Proportion of environmental surveillance (ES) samples at each site with enterovirus detection grouped by state. Sites are labeled with an arbitrary letter for clarity of display and the number of samples collected at that site indicated in brackets. Error bars indicate 95% confidence intervals. FCT, Federal Capital Territory.

One thousand three hundred forty-five ES samples were collected from sites included in this study between June 1, 2018 and May 31, 2019. The median number of samples collected from a site was 12 (ie, monthly) and ranged from 9 to 49 (IQR, 11–24). The prevalence of enterovirus isolation, defined as the proportion of samples tested at a site that were positive for any enterovirus (including poliovirus), varied between 9% and 100% (mean 68%) among ES sites (Figure 3). The prevalence of Sabin poliovirus varied between 0% and 68% (mean 26%) across sites, and serotype 2 VDPV was detected in 67 samples from 22 sites (no other serotype of VDPV was detected). Nineteen (37%) ES sites detected enterovirus in >80% of samples, 41 (53%) in >70% of samples, and 61 (78%) in >50% of samples.

**Figure 4.**
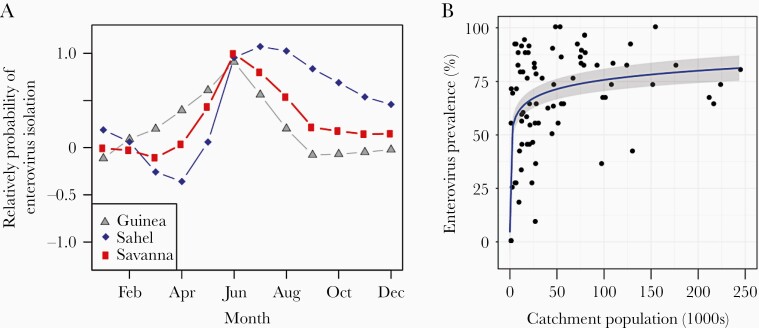
Variables associated with the prevalence of enterovirus detection at environmental surveillance sites include (A) month and (B) estimated catchment population based on digital elevation models. In A, the relative probability of enterovirus detection on a logit scale is shown, as estimated by the random effect of the logistic regression model without any fixed effects included. In B, the prevalence of enterovirus detection is shown against catchment population based on DEM/GRID3 estimates together with the predicted mean (line) and 95% confidence interval (gray area) based on a linear regression on the log(population) scale.

In the mixed-effects logistic regression, the monthly trend in enterovirus detection estimated by the cyclical random walk was strongly seasonal showing a peak in June in the Savanna and Guinea climatic zones and a somewhat later peak in July in the northern Sahel zone (Figure 4). The association of ES site characteristics with detection of enterovirus (poliovirus or NPEV) is shown in Table 2. In the univariable analysis, several water quality parameters were associated with enterovirus detection including higher temperature (≥27°C vs <22°C), pH (≥8.5 vs <7.5), and TDS (≥434 vs <434 mg/L). A larger catchment population was also significantly associated with enterovirus prevalence when based on DEM/GRID3 estimates or WorldPop population within 2 km but not when based on estimates provided by ES officers. The relationship between the catchment population based on DEM/GRID3 and the prevalence of enterovirus detection is shown in Figure 4. The final multivariable model with the lowest WAIC included DEM/GRID3 catchment population estimates, as well as pH, TDS, and specimen volume (WAIC =1 437.87) (Table 2).

**Table 2.** Univariable and Final Multivariable Mixed-Effects Logistic Regression Model of Enterovirus Detection in ES Samples

Variable	Level	Univariable Odds Ratio [95% CI]	Multivariable Model Odds Ratio [95% CI]
Water Quality Parameters			
Temperature (°C)	<21.8	Ref	
	21.8–27.1	0.88 [0.66–1.19]	
	≥27.1	1.67 [1.12–2.45]	
pH	<7.5	Ref	Ref
	7.5–8.5	1.22 [0.93–1.6]	1.13 [0.86–1.49]
	≥8.5	2.2 [1.05–4.82]	2.17 [1.04–4.73]
Oxidative reductive potential (mV)	−197.8 to 77.2	Ref	
	<−197.8	1.29 [0.93–1.78]	
	≥77.2	1.13 [0.79–1.61]	
Dissolved oxygen (% saturation)	<38	Ref	
	38–74.9	1.07 [0.81–1.41]	
	≥74.9	1.25 [0.85–1.82]	
TDS (mg/L)	<434.2	Ref	Ref
	434.2–1170	1.34 [1–1.8]	1.34 [0.99–1.80]
	≥1170	1.75 [1.2–2.55]	1.77 [1.21–2.58]
Turbidity (NTU)	<12.1	Ref	
	12.1–61.2	1.4 [1.07–1.83]	
	≥61.2	1.55 [1.08–2.22]	
Catchment Population Estimates			
Population within 2 km based on WorldPop	<50 k	Ref	
	50–100 k	1.31 [0.92–1.85]	
	≥100 k	1.99 [1.35–2.93]	
ES Officer estimate	<50 k	Ref	
	50–100 k	1.39 [0.75–2.58]	
	≥100 k	1.09 [0.79–1.52]	
Population based on DEM and GRID3 data	<12 500	Ref	Ref
	12 500–75 k	1.50 [1.08–2.08]	1.45 [1.04–2.00]
	≥75k	2.12 [1.38–3.26]	2.22 [1.45–3.37]
Field Team Survey			
Sewage smell	No	Ref	
	Yes	1.2 [0.9–1.6]	
Sewage depth	Deep	Ref	
	Medium	1.03 [0.75–1.42]	
	Shallow	0.9 [0.57–1.43]	
	Unclear	1.2 [0.64–2.3]	
Speed of sewage flow	Fast	Ref	
	Moderate	1.0 [0.75,1.32]	
	Slow	1.26 [0.89–1.80]	
	Stagnant	1.09 [0.32–3.85]	
Laboratory Data			
Time of sample collection	6–8 am	Ref	
	After 8 am	0.44 [0.03–6.55]	
	Before 6 am	1.88 [0.89–4.11]	
Temperature of sample carrier (°C)	<6°C	Ref	
	≥6°C	0.76 [0.42–1.4]	
Sample condition	Good	Ref	
	Bad	0.45 [0.13–1.58]	
Sample volume (L)	<1	Ref	Ref
	>1	0.85 [0.66–1.08]	0.78 [0.61–1.00]
Time from collection to arrival in laboratory	0–1 day	Ref	
	2 or more days	1.55 [0.82–3.05]	
Time from arrival in laboratory to processing	<7 days	Ref	
	≥21 days	1.77 [0.49–7.57]	
	7–20 days	0.88 [0.55–1.42]	
Volume of sewage concentrate (mL)	10–15	Ref	
	15+	0.88 [0.68–1.14]	
	<10	0.61 [0.21–1.8]	
Facilities Within a 10-Minute Walk (ES Officer Survey)			
School	No	Ref	
	Yes	1.08 [0.78–1.49]	
Hospital/health facility	No	Ref	
	Yes	1.2 [0.79–1.84]	
Factory	No	Ref	
	Yes	0.91 [0.53–1.57]	
Transit or commercial hub	No	Ref	
	Yes	1.19 [0.87–1.63]	

Abbreviations: CI, confidence interval; DEM, digital elevation models; ES, environmental surveillance; mV, millivolts; NTU, nephelometric turbidity units; Ref, reference category; TDS, total dissolved solids.

### Machine Learning Prediction of Environmental Surveillance Site Performance

**Figure 5.**
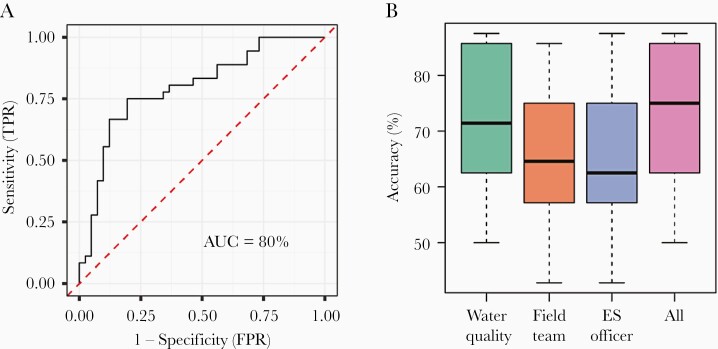
Machine learning (random forests) prediction of environmental surveillance (ES) site performance as good (>70% enterovirus isolation in ES samples) or bad (≤70% enterovirus). In A, the receiver operator characteristic curve for prediction of the observed data is shown for a best-fit random forest model. In B, the out-of-sample predictive accuracy of random forests for 20 repetitions of 10-fold cross-validation is shown (ie, leaving out 10% of ES sites for each model fit and predicting their performance based of the model fit to the other sites). The bars indicate the interquartile range of the out-of-sample model accuracy, the central line indicates the median, and the whiskers indicate the 95% intervals. Results are shown for the models based on water quality parameters, field team survey data, ES officer data (including catchment population estimates), and all data combined. AUC, area under the curve.

The fit of a single random forests model to the aggregated ES site characteristic data gave an area under the receiver operator characteristic (ROC) curve of 80% indicating reasonable accuracy in correctly classifying ES sites as good (>70% enterovirus isolation) or bad (≤70%) (Figure 5). The curve indicates that the model is able to predict good ES sites with approximately 75% sensitivity and specificity. When fitting multiple random forests models to data from 90% of ES sites and performing out-of-sample predictions for the remaining 10% (ie, 10-fold cross-validation), the median predictive accuracy was 75% (IQR, 63%–86%) when using water quality, ES officer (including catchment population), and field team data combined (Figure 5). Most information came from the water quality data, which alone gave a median out-of-sample predictive accuracy of 71% (IQR, 63%–86%). The most important variables based on their contribution to the Gini coefficient were the maximum TDS recorded at the site (across the 4 visits), population within 2 km, and the minimum ORP. A model based on a single measurement of ES site characteristics (Quarter 1 data) gave the same predictive accuracy (median, 75%; IQR, 63%–86%).

## Discussion

The prevalence of enterovirus detection including poliovirus and NPEV in ES samples is routinely used as an indicator of ES site sensitivity to detect poliovirus circulation. In Nigeria, 41 of 78 ES sites detected enteroviruses in >70% samples and 67 serotype 2 VDPV were isolated during the study period (compared with 34 serotype 2 VDPV AFP cases in the same states), indicating a sensitive ES system. Nonetheless, 17 (22%) sites detected enteroviruses in less than 50% of samples, suggesting that ES sensitivity could be further improved. In other countries in Africa, the prevalence of enterovirus detection has been considerably lower, further indicating the need for improved guidelines and implementation of ES site selection (eg, all 12 sites reported in [24] in Cameroon had <50% enterovirus prevalence during 2016–2017).

In this study, easily measured water quality parameters correlated with enterovirus isolation in ES samples and gave 75% out-of-sample accuracy to predict good versus bad ES sites. Total dissolved solids and pH were included in the final multivariable logistic regression model for enterovirus detection in ES samples, and TDS was also the most important classifier in the random forests model of site performance. Total dissolved solids includes both organic and inorganic substances and is a widely used measure of water quality that may increase as a result of fecal contamination, but also other processes such as agricultural runoff. Indeed, TDS measured in quarter 1 was significantly correlated with the number of people living within 2 km of the ES site (r = 0.268, *P = .*0179) (Supplementary Figure 1), consistent with its role as a measure of the extent of fecal contamination. However, both TDS and catchment population were included in the final regression model, suggesting they are independently associated with enterovirus detection (TDS did not correlate with catchment based on DEM/GRID3 or ES officer survey) (Supplementary Figure 1). In addition, TDS can promote poliovirus adsorption to solid waste components, which may increase poliovirus survival and therefore detection by cell culture [25]. The association of acidic pH with lower enterovirus prevalence may reflect poliovirus inactivation in sewage or wastewater contaminated by factory or industrial effluents. Although poliovirus is stable at a range of pH values, its survival is reduced at extreme pH values that might occur in the case of industrial pollution [25].

Enterovirus prevalence was strongly associated with ES site catchment population estimated using DEM/GRID3 or WorldPop population data but not when estimated by ES officers using vaccination microplans or census data. This suggests that publicly available population data such as WorldPop could be used to help with initial selection of site placement when beginning or expanding poliovirus ES. More detailed planning could then be facilitated by DEM using synthetic or field collected data to demarcate the catchment area—an important consideration when targeting specific high-risk neighborhoods or avoiding overlapping catchments for closely located sites. It is unclear why catchment population estimates from ES officers were larger than DEM/GRID3 estimates, although this may reflect expectations based on WHO guidelines to choose sites with a catchment of 100 000 to 300 000, which is considerably larger than DEM/GRID3 estimates for the majority of sites.

Enteroviruses were slightly more prevalent when a smaller sample volume was collected (<1 liter). We speculate that this may reflect an effort by ES officers to collect a larger sample volume when they judge the sewage to be too dilute to allow poliovirus detection.

Our study had a number of limitations. Although we were able to quantify key sewage water quality parameters, other measures such as flow speed, depth, and their daily fluctuations were described by subjective categories that may limit comparability between ES sites visited by different teams. Future studies could aim to more accurately quantify these site characteristics using appropriate technology. We also report results from only a single country. To determine whether our findings hold in other settings, it will be important to measure ES site characteristics in other countries, particularly those with lower rates of enterovirus detection. Given the retention of predictive accuracy in the random forests model with data from just a single visit to each ES site, assessment in other countries could be rapid and focus on the key parameters that we have identified in Nigeria (ie, TDS, pH, and catchment population). Finally, we used the prevalence of enterovirus isolation on human RD cells as an indicator of human fecal contamination and a proxy of ES site sensitivity. We found that increased catchment population size increased the probability of enterovirus detection. However, single or small numbers of poliovirus infections will shed a limited amount of virus, and this may be diluted to undetectable levels in sewage from large catchment populations [10]. Therefore, large populations may require more than 1 ES site or more frequent sampling to ensure adequate sensitivity to detect low prevalence poliovirus infections. In areas with circulating polioviruses, detection of these viruses in ES compared with AFP surveillance, and the genetic divergence of each isolate from other detected viruses, can give an indication of ES sensitivity [3, 4]. Analysis of these data in relation to ES site characteristics may help further optimize ES by identifying site or system characteristics important for detection of low prevalence polioviruses.

## Conclusions

If our findings are replicated in other countries, we suggest that the specific and measurable ES site characteristics we have identified should be incorporated into WHO guidelines for the establishment of new ES sites in countries supported by the GPEI. This would facilitate more timely and sensitive poliovirus ES during planned expansion and in response to outbreaks.

## Supplementary Data

Supplementary materials are available at *The Journal of Infectious Diseases* online. Consisting of data provided by the authors to benefit the reader, the posted materials are not copyedited and are the sole responsibility of the authors, so questions or comments should be addressed to the corresponding author.

jiaa175_suppl_Supplementary_Figure_1

## Notes

***Financial support.*** This work was funded by the Bill & Melinda Gates Foundation (OPP1171890).

***Potential conflicts of interest.*** All authors: No reported conflicts of interest. All authors have submitted the ICMJE Form for Disclosure of Potential Conflicts of Interest.

Presented in part: Global Polio Eradication Initiative, Environmental Surveillance Implementation Working Group, November 2019, Geneva, Switzerland.
