# Supplementary material for: Characterizing Environmental Surveillance Sites in Nigeria and Their Sensitivity to Detect Poliovirus and Other Enteroviruses
Source: J Infect Dis. 2020 Apr 9;225(8):1377–86. doi: 10.1093/infdis/jiaa175 (PMC9016446; doi:10.1093/infdis/jiaa175)
Supplement: jiaa175_suppl_Supplementary_Figure_1 [file jiaa175_suppl_supplementary_figure_1.pdf]

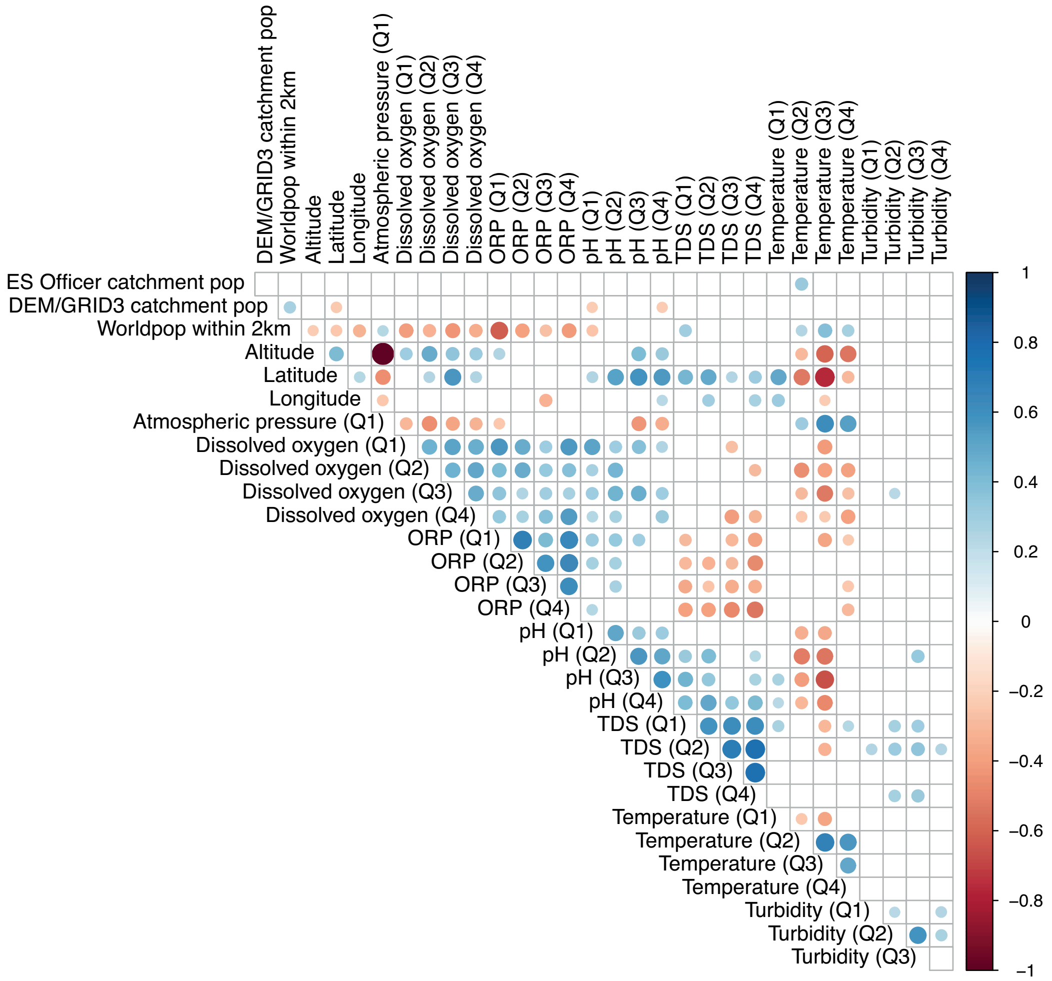

Supplementary Figure 1 Correlation between catchment population estimates and measurements taken by the field team at 78 ES sites. The size and colour of the circles is determined by the Pearson correlation coefficient and only significant correlations are shown (P<0.05). The measurement quarter is indicated by Q1 etc.
